# FAMS—A Targeted Fatty Acid Mass Spectrometry Method for Monitoring Free Fatty Acids from Polysorbate Hydrolysis

**DOI:** 10.3390/mps7050071

**Published:** 2024-09-07

**Authors:** Anja Bathke, Sina Hoelterhoff, Jan Wendler, Inn H. Yuk, Christian H. Bell

**Affiliations:** 1 Pharma Technical Development, F. Hoffmann-La Roche, Grenzacherstrasse 124, 4070 Basel, Switzerlandjan.wendler@roche.com (J.W.);; 2 Pharma Technical Development, Genentech, 1 DNA Way, South San Francisco, CA 94080, USA

**Keywords:** liquid chromatography, mass spectrometry, single quad, polysorbate degradation, free fatty acid, biopharmaceuticals, automation, high-throughput, method validation

## Abstract

Polysorbates are the predominant surfactants used to stabilize protein formulations. Unfortunately, polysorbates can undergo hydrolytic degradation, which releases fatty acids that can accumulate to form visible particles. The detection and quantitation of these fatty acid degradation products are critical for assessing the extent of polysorbate degradation and the associated risks of particle formation. We previously developed a user-friendly mass spectrometric method called Fatty Acids by Mass Spectrometry (FAMS) to quantify the free fatty acids. The FAMS method was validated according to ICH Q2 (R1) guidelines and is suitable for a wide range of products, buffers and protein concentrations. The end-to-end workflow can be automated from sample preparation to data analysis. To broaden method accessibility, the QDa detector selected for fatty acid measurement does not require specific mass spectrometry experience. We provide here a detailed procedure for both manual and automated sample preparation for high-throughput analysis. In addition, we highlight in this protocol the critical operational details, procedural watchouts and troubleshooting tips to support the successful execution of this method in another laboratory.

## 1. Introduction

Polysorbates are non-ionic surface-active compounds (surfactants) frequently added to stabilize and protect the protein biotherapeutic over the shelf-life of the drug product [1,2,3,4,5]. In this context, polysorbate 20 (PS20) and polysorbate 80 (PS80) are the most commonly used surfactants in registered pharmaceuticals spanning a variety of administration forms [6]. Despite their excellent stabilizing properties, polysorbates themselves can be unstable and degrade via two pathways—oxidation and hydrolysis [5,6,7,8,9,10,11,12]. 

Oxidative degradation can be triggered by various factors such as light, temperature, oxygen exposure and residual metals. The primary indicators for polysorbate (PS) oxidation are the presence of aldehydes, ketones, peroxides and short-chain esterified polyoxyethylene (POE) sorbitan/isosorbide species [7]. 

While PSs can undergo hydrolysis at the carboxylic ester bond via chemical or enzymatic routes, chemical hydrolysis is unlikely under pharmaceutically relevant conditions [10], and enzymatic hydrolysis has been established as the primary mechanism for PS degradation [8]. Unlike oxidation, hydrolysis leads exclusively to the accumulation of free fatty acids (FFAs) and non-esterified POE sorbitan/isosorbide species [5,9,11]. 

The acceptable ranges for fatty acid ester compositions of PS20 and PS80 are described in various pharmacopeias, such as the Chinese Pharmacopoeia (ChP), European Pharmacopoeia (Ph. Eur), Japanese Pharmacopoeia (JP) and United States Pharmacopoeia (USP). Depending on the type of PS, the degradation profile and the resulting composition of PS degradants (i.e., FFAs of different chain lengths) can vary considerably. For example, the degradation of PS20 releases mostly lauric acid (LA) and myristic acid (MA), whereas the degradation of PS80 releases mostly oleic acid (OA) [13,14]. 

The degradation of PS in the drug product increases the risk of protein aggregation and degradation at interfaces (e.g., air/liquid) from inadequate surfactant protection. It is important to assess the risk of increased immunogenicity from protein aggregation [15,16,17], while meeting the requirements of the pharmacopeias [18]. Hydrolytic PS degradation presents an additional challenge in the form of particle risks—the accumulation of FFAs generated can result in their precipitation as visible particles when the FFAs exceed their respective solubility limits. To assess the risk of FFA particle formation in drug products, Doshi et al. updated an FFA solubility model for PS20 formulations [19,20]. They demonstrated that the solubilities for the longer-chain free fatty acids (FFAs) such as MA, palmitic acid (PA) and stearic acid (SA) are lower than for the most abundant LA. Therefore, these longer-chain FFAs are more prone to precipitate and form visible and subvisible particles [19]. Correspondingly, it would be important to quantify the amount of FFAs to assess the risk of particle formation.

A variety of methods have been developed to quantify the extent of PS hydrolytic degradation and, therefore, evaluate the associated particle risks [12,13,21,22,23]. In recent years, our laboratory (Honemann et al. and Hoelterhoff et al.) developed a mass spectrometric method for the quantification of FFAs, referred to as Fatty Acids by Mass Spectrometry (FAMS). The FAMS method was validated according to ICH Q2 (R1) guidelines initially for LA, MA and OA [22] and then extended to include the longer-chain fatty acids PA and SA [21]. We also extended the FAMS method for high-throughput analysis by implementing automation through the use of robotic platforms and preparation techniques. This protocol provides the detailed procedure for both manual and automated sample preparation using the FAMS method previously established in our laboratory [21,22] and further includes additional operational details, procedural watchouts and troubleshooting tips that were not previously disclosed.

## 2. Experimental Design

### 2.1. Equipment

- Analytical balance (Mettler Toledo XPE205DR)
- Precision pipettes (e.g., Gilson pipettes covering 2 to 2000 µL with appropriate tips)
- Ultra-Pure Water System (Millipore Milli-Q Advantage A10)
- ACQUITY UPLC H-Class (plus) System from Waters Corporation (Milford, MA, USA) connected to a QDa mass spectrometer (QDa) from Waters
- ACQUITY UPLC column in-line filter as pre-column to a Jupiter^®^ C4 RP column (300 Å, 2 × 50 mm, 5 μm) from Phenomenex (Torrance, CA, USA)

### 2.2. Manual Sample Preparation

- Glass vials for precipitation reagent: Amber vials 2 mL (Supelco, Cat. No. 27000, Bellefonte, PA, USA)
- 1.5 mL Eppendorf Safe-Lock Tubes, US Cat. No.: 022363204 or EU Cat. No.: 0030120.086
- HPLC vials (300 µL Fixed Insert Vial (Clear, Screw Top), Thermo Scientific, Cat. No. 03-FISV, Langerwehe, Germany)
- HPLC caps for vials (VWR, PP Screw cap 9 mm, blue, Silic. whi./PTFE blue, slitted, 55°, 1.0 mm, Cat. No. 548-0088, Leuven, Belgium)

### 2.3. Automated Sample Preparation

1. Robotic system
  - Hamilton Microlab STAR workstation
  - Other systems can be used as well (e.g., Tecan Fluent^®^ 1080, Tecan Freedom EVO200^®^, Maennedorf, Switzerland)
2. Positive pressure module
  - e.g., [MPE]^2^ (Hamilton)
  - e.g., Resolvex^®^ (Tecan)

- Shaker: HHS 3.0 MTP FLAT BOTTOM (Hamilton, Cat. No. 199034, Bonaduz, Switzerland)
- Glass vials for precipitation reagent: Amber vials 5 mL (Supelco, Cat. No. INFCG075Y-14/050-D, Bellefonte, PA, USA)
- PierceTM Protein precipitation plate (ThermoFisher Scientific, Cat. No. 90036 or 90037, Waltham, MA, USA)
- A 96-well collection plate with 700 µL glass inserts (Waters Corporation, Cat No. 186000349, Milford, MA, USA)
- Cap-mat 96-well Square Plug Pre-slit Silicone/PTFE (Waters Corporation, Cat. No. 186006335, Milford, MA, USA)

### 2.4. Reagents

- Stable isotopically labeled internal standards:
  − Lauric acid D23 (Sigma Aldrich, Cat. No. 451401, St. Louis, MO, USA)
  − Myristic acid 13C14 (Sigma Aldrich, Cat. No. 605689, St. Louis, MO, USA) or Myristic acid D27 (Sigma Aldrich, Cat No. 68698, St. Louis, MO, USA)
  − Palmitic acid D31 (Sigma Aldrich, Cat No. 366897, St. Louis, MO, USA)
  − Stearic acid 13C18 (Sigma Aldrich, Cat No. 605581, St. Louis, MO, USA)
  − Oleic acid 13C18 (Sigma Aldrich, Cat. No. 490431, St. Louis, MO, USA)
- Ammonium acetate (Sigma Aldrich, Cat. No. 73594, St. Louis, MO, USA)
- Methanol LiChrosolv (Merck, Cat. No. 1.06007.2500, Darmstadt, Germany)
- Acetone puriss, p.a. (Sigma Aldrich, Cat. No. 32201, St. Louis, MO, USA)

### 2.5. Reagent Setup

- Mobile phase A (20 mM ammonium acetate)
- To a 1 L volumetric flask, add 1.54 g ammonium acetate, adjust to volume with Milli-Q water, and stir until completely dissolved. Filter mobile phase with 0.2 µm filter.
- Mobile phase B (100% methanol)
- Precipitation stock (80:20 acetone:methanol)

Mix 240 mL of acetone with 60 mL of methanol.

Preparation of internal standards (stock solutions used for precipitation reagents)

- Lauric acid (LA) D23 Stock

Dissolve 50 mg of lauric acid D23 in 50 mL precipitation stock.

- Myristic acid (MA) D27 Stock

Dissolve 50 mg of myristic acid D27 in 50 mL precipitation stock.

- Palmitic acid (PA) D31 Stock

Dissolve 50 mg of palmitic acid D31 in 50 mL precipitation stock.

- Stearic acid (SA) 13C18 Stock

Dissolve 50 mg of stearic acid 13C18 in 50 mL precipitation stock.

- Oleic acid (OA) 13C18 Stock

Dissolve 50 mg of oleic acid 13C18 in 50 mL precipitation stock. 

Use one of the following precipitation reagents

- Precipitation reagent (1 µg/mL per FFA) for PS20 formulations (to quantify LA, MA, PA and SA)
- Dilute 200 µL of lauric acid D23, myristic acid D27, palmitic acid D31 and stearic acid 13C18 stocks and fill up to 200 mL with precipitation stock to yield a final concentration of 1 µg/mL per FFA. Aliquots can be stored up to two years in glass vials at −80 °C.
- Precipitation reagent (1 µg/mL per FFA) for PS80 formulations (to quantify OA, PA and SA)
- Dilute 200 µL of oleic acid 13C18, palmitic acid D31 and stearic acid 13C18 stocks and fill up to 200 mL with precipitation stock to yield a final concentration of 1 µg/mL per FFA. Aliquots can be stored up to two years in glass vials at −80 °C.

Note: Recipes are for nominal quantities of reagent and can be adjusted proportionally according to assay requirements.

## 3. Procedure

During sample preparation, it is important to avoid contamination with fatty acids from external sources. For example, fatty acids may be unintentionally introduced through using consumables that are not suitable (see Box 1—TIPS AND TRICKS).

Box 1 **Tips and Tricks.**

1. During sample preparation, it is essential to ensure that the pipette tip does not reach the pellet, such that the protein pellet is destroyed and then possibly drawn on.
2. For easier handling, the precipitation reagent with the labeled standard can be aliquoted and stored at −70 °C for 26 months.
3. Samples with a FFA content greater than 40 µg/mL should be diluted prior sample preparation (reportable range for FFAs lower than 40 µg/mL).
4. Prepared samples can be stored in the HPLC- vial or 96-well plate with glass inserts for future analysis at 20 °C for up to 72 h.
5. Equilibrate the column for at least 12 min at starting conditions prior to the first injection.
6. Run wash gradient 3 times after each sequence.
7. To avoid fatty acid contamination, be particularly attentive to the selection of consumables [23] and gloves (do not wear HALYARD—purple nitrile powder free EXAM gloves). Also, do not use any kind of hand cream prior sample preparation.

### 3.1. Manual Sample Preparation

First, add 50 µL of the protein solution to a 1.5 mL Eppendorf tube (refer to EQUIPMENT for detailed information). Next, add 200 µL of the respective precipitation reagent with a pre-wetted pipette tip. In this manner, 1 volume of protein solution is combined with 4 volumes of precipitation reagent. Mix by vortexing the tube and then incubate at room temperature for 1 h to allow the protein to precipitate. Spin down the precipitate by centrifugation (15,000× *g* for 15 min at 20 °C). After the centrifugation step, the resulting pellet can differ in size and appearance (see Box 2—Protein precipitation).

Box 2 **Protein precipitation.**

Depending on the protein concentration and the buffer composition, the pellets can differ in size and in appearance.

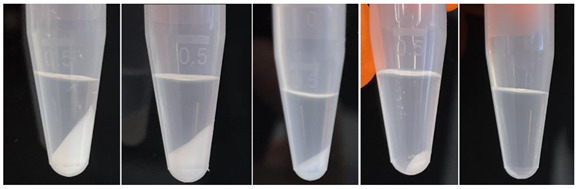

Furthermore, the composition of the precipitation reagent in respect to the labeled FAs can vary.

This means that only the labeled FAs that need to be analyzed have to be added to the precipitation reagent.

**Figure 1:**
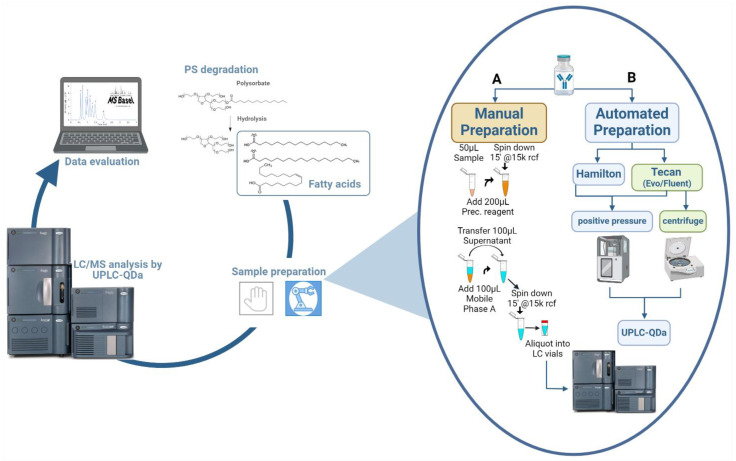
Overview of LC/MS method for the quantification of free fatty acids in biopharmaceuticals and the two different procedures of the sample preparation. (**A**) Procedure for the manual sample preparation and (**B**) for high-throughput application by using robotic platforms (created with BioRender.com, accessed on 7 June 2024). Figure adapted from Hoelterhoff et al. [21].

Add 100 µL of mobile phase A into a fresh 1.5 mL Eppendorf tube (refer to EQUIPMENT for detailed information), add 100 µL of the supernatant and mix by vortexing the tube. Spin down by centrifugation (15,000× *g* for 15 min at 20 °C). Transfer 50–150 µL of this mixture of mobile phase A and supernatant to LC-MS-vials (see Figure 1A) [21,22].

### 3.2. Automated Sample Preparation

First, add 1 mL methanol to each well to prewash the Pierce^TM^ protein precipitation plate wells. This step can be performed using either a centrifuge (500× *g* for 7 min at room temperature in Eppendorf Centrifuge 5804) or the [MPE]^2^ positive pressure manifold (18 psi four times for one minute). Store methanol in a glass container or a rust-proof metal trough.

Next, use the robotic system to add 50 µL of the antibody solution and 200 µL of the respective precipitation reagent to the prewashed filter membrane of the Pierce protein precipitation plate. Then, incubate or shake the plate at room temperature for 5 min at 600 rpm on the shaker. 

Filter the sample mixture into a 96-well collection plate (loaded with 700 µL glass inserts). This step can be performed by centrifugation (1200× *g* for 15 min at room temperature) with an Eppendorf Centrifuge 5804) or by [MPE]^2^ positive pressure manifold (18 psi four times for 2.5 min). 

Add 100 µL of mobile phase A and 100 µL of the supernatant to a new 96-well collection plate (loaded with 700 µL glass inserts). 

Incubate or shake the collection plate at room temperature for 5 min at 800 rpm on the shaker. (see Figure 1B) [21]. Seal the plate with a cap-mat.

### 3.3. Data Acquisition by LC/MS

Perform LC/MS with an ACQUITY UPLC H-Class System (Waters Corporation, Milford, MA, USA) that is equipped with autosampler and column compartment under temperature control. 

Use a Jupiter^®^ C4 RP column from Phenomenex (Torrance, CA, USA) to perform liquid chromatography (LC). The chromatographic separation of FFAs was achieved with 20 mM ammonium acetate as mobile phase A and methanol as mobile phase B (Table 1) [21]. FFAs can also be separated under isocratic conditions (35% mobile phase A) for 5 min at a flow rate of 0.4 mL/min [22]. 

Maintain autosampler at 20 °C and column compartment at 60 °C. Set the injection volume to 8 μL. 

For FFA detection, use a connected QDa Performance mass spectrometer (Waters) equipped with an external backing pump in negative ion mode. 

Apply the following mass spectrometry (MS) settings: cone voltage 15 V; source temperature 120 °C; capillary voltage 800 V; probe temperature 600 °C; mass range 50–1000 m/z; and sampling frequency 2 Hz [21,22]. 

For MS measurements, extract the masses listed in Table 2 for PS80 samples and in Table 3 for PS20 samples.

Upon completion of MS measurements, rinse the column first with the wash gradient (Table 4) and then with the storage gradient (Table 5). 

### 3.4. Data Evaluation

Evaluate the data using TargetLynx via MassLynx Software Version 4.1 (SCN781) or Empower (Waters Corporation, Milford, MA, USA). Quantify the FFA of interest (i.e., LA, MA, OA, PA or SA) by comparing the peak area of the FFA with its internal labeled standard using the formula:

$$Conc.\left(FFA\right)=\frac{\sum Peak\;areas\;of\;FFA}{\sum Peak\;areas\;of\;internal\;standard}*4*1\;\frac{µg}{mL}$$

The ∑Peak areas of FFA refers to the sum of the monoisotopic peak and the isotopic peaks at +1/+2 or −1/−2 of free fatty acids. 

The ∑Peak areas of Internal standard refers to the sum of the monoisotopic peak and the isotopic peaks at +1/+2 or −1/−2 of labeled fatty acids. 

**Figure 2:**
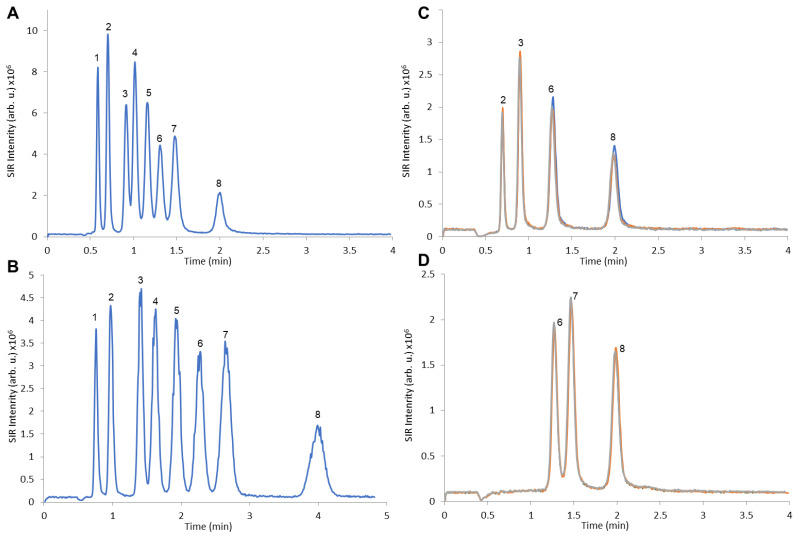
Single-ion-recordings (SIRs) and retention times of FFAs (1: caproic acid C10, 2: lauric acid C12, 3: myristic acid C14, 4: palmitoleic acid C16:1, 5: linoleic acid C18:2, 6: palmitic acid C16, 7: oleic acid C18:1 and 8: stearic acid C18). (**A**) Summed SIRs of a FFA mixture comprising the most prevalent FAs in PS20 and PS80 using linear gradient. (**B**) Summed SIRs of a FFA mixture comprising the most prevalent FAs in PS20 and PS80 using isocratic separation. (**C**,**D**) Overlays of the SIRs with linear gradient of lauric (2), myristic (3), palmitic (6), oleic (7) and stearic acid (8) of injection #1 (marked in blue), #50 (marked in orange) and #100 (marked in grey) in formulated drug product with PS20 (**C**) and PS80 (**D**).

The FAMS method described in this protocol allows for the baseline separation of a mixture of multiple FFAs (such as those typically observed after hydrolysis of PS20 and PS80). The separation is completed within 5 min via isocratic separation (Figure 2B) [22] and within 4 min via the linear gradient (Figure 2A) [21].

To test the robustness of this LC method, a PS20 formulated sample and a PS80 formulated sample were each injected sequentially 100 times. The SIRs obtained for LA, MA, PA and SA from the PS20 sample (Figure 2C) or correspondingly PA, SA and OA from the PS80 sample (Figure 2D) were visually compared. The results from injections 1, 50 and 100 overlaid in Figure 2C,D demonstrate high reproducibility.

In general, a system suitability test (SST) sample should be injected in parallel for every measurement. For SST purposes, choose samples with a low and stable content of free fatty acids over time covering either PS20 or PS80 containing protein formulations. The UHPLC-QDa system can thus be assessed if it is operating as intended (and hence if the results are valid) by comparing the SST sample measurements against the predefined SST criterion [21] (refer to TROUBLESHOOTING section under RESULTS).

## 4. Expected Results

### 4.1. Method Validation

As previously described in detail, FAMS method validation was performed for both the manual [22] and automatic sample preparation [21] and for the isocratic [22] and linear separation [21]. Three recombinant monoclonal antibodies (mAbs) in PS20 or PS80 formulation at different protein concentrations (ranging from 50 mg/mL to 183 mg/mL) were used to support the FAMS method validation [21].

The various parameters tested as part of the FAMS method validation are listed in Table 6.

### 4.2. Troubleshooting

In the event that the predefined system suitability test criterion is not met (e.g., the content of free fatty acids of the SST sample is not within a defined range), the sample preparation process should be examined, and the UHPLC-QDa system should be checked for the pressure during analysis. The UHPLC-QDa problem may be solved by either exchanging the ESI-Probe and/or the aperture disc, changing the column if needed (e.g., high pressure or poor resolution) or cleaning the QDa source by using a cleaning solution (50:50 IPA: purified water, 0.1% formic acid). 

**Figure 3:**
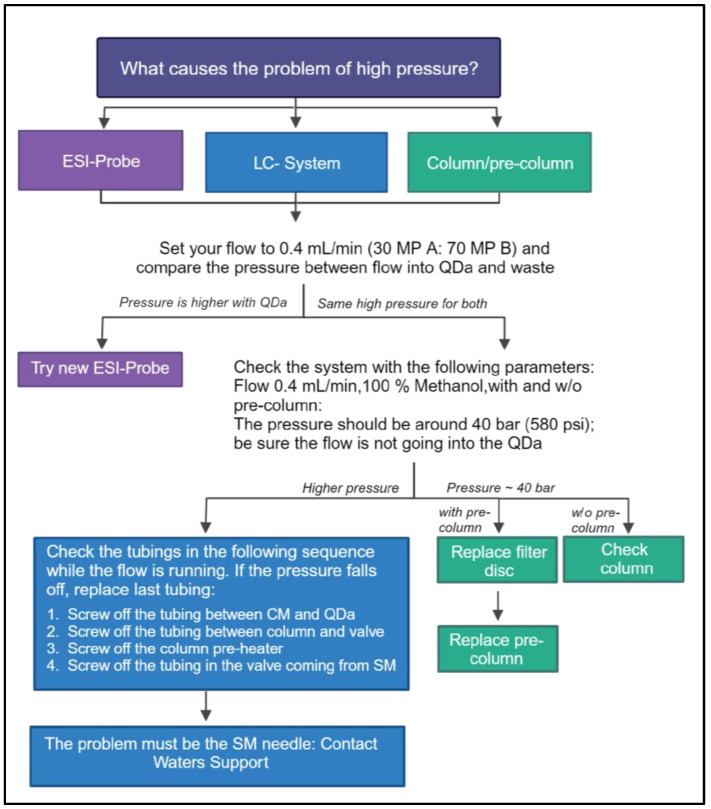
Troubleshooting instructions if system pressure is observed to be too high on the LC system (created with BioRender.com, accessed on 28 May 2024).

In the event that the UHPLC-QDa system pressure is too high, the instructions in Figure 3 must be followed. 

If the total ion chromatogram is very noisy, the peaks of SIRs are jagged or retention time shifts are observed, and both the LC system and the Sample Manager are purged with (25:25:25:25 IPA: ACN: MeOH: purified water, 0.1% formic acid). After purging, a flow rate of 0.1 mL/min (without column) is set for an overnight cleaning procedure for the LC system.

## 5. Discussion and Conclusions

The validation results demonstrate the suitability of this FAMS method for multi-product use. This method is valid for the use of multiple sample types ranging from in-process samples to drug products, provided in various buffer systems and at different protein concentrations.

Implementing two different procedures of sample preparation, manual and automated on varying robotic platforms, allows users to choose the workflow that best suits their needs. Here, the sample number is only limited by the storage time in the autosampler (72 h). The results also show that both linear and isocratic gradients are suitable for FFA separation via LC. 

From a technical perspective, a single quad mass spectrometer, i.e., the QDa system, is utilized. This kind of MS detector enables a user-friendly handling and does not require specific MS experience to operate, thus making the system amenable for broader use. Nevertheless, other mass spectrometers (single quad or high-resolution) from different vendors can be suitable as well. An important aspect of our work is the transferability of this method in a GMP-regulated environment. The full qualification ability of the UHPLC-QDa system (in terms of hardware and software) can support the use of this method under GMP regulations. 

As previously described, we use a web application to generate an Excel report that includes all the information collected and the calculations used, simplifying data treatment [21].

A key part of FAMS that needs to be taken into account is the contamination of free fatty acids from external sources such as consumables and equipment. Selection, testing and handling of appropriate material is crucial, and analysts must be trained accordingly. Furthermore, it is recommended to check the validated parameters before transferring the FAMS method. Even if the validation has been described previously and suits our purpose of monitoring free fatty acids, the limits of the methods should be evaluated and adapted to the respective application and its requirements.

In summary, with the highly automated setup described here, we have developed an efficient end-to-end process—from sample preparation to data analysis to the reporting of results for FFA quantitation—to support PS degradation investigations. 

## Figures and Tables

**Table 1:** Gradient for chromatographic separation of FFAs [21].

Time [min]	Mobile Phase A (20 mM Ammonium Acetate) [%]	Mobile Phase B (Methanol) [%]
0.0	30.0	70.0
0.5	30.0	70.0
3.4	15.0	85.0
3.5	30.0	70.0
4.0	30.0	70.0

**Table 2:** Extracted masses of free and labeled (*) fatty acids for MS measurements for PS80 samples. Monoisotopic peaks and the most abundant isotopic peaks at +1/+2 or −1/−2 are listed.

Channel	Name Of Fatty Acid	Extracted Masses (m/z)
1	Palmitic acid (PA)	255.2310
2	PA +1	256.2350
3	PA D31 * −1	285.4220
4	PA D31 *	286.4250
5	Oleic acid (OA)	281.2460
6	OA +1	282.2510
7	OA 13C18 * −1	298.3030
8	OA 13C18 *	299.3070
9	Stearic acid (SA)	283.2620
10	SA +1	284.2670
11	SA 13C18 * −1	300.3190
12	SA 13C18 *	301.3220

**Table 3:** Extracted masses of free and labeled (*) fatty acids for MS measurements for PS20 samples. Monoisotopic peaks and the most abundant isotopic peaks at +1/+2 or −1/−2 are listed.

Channel	Name of Fatty Acid	Extracted Masses (m/z)
1	Lauric acid (LA)	199.1680
2	LA+1	200.1730
3	LA+2	201.1780
4	LA D23 * −2	220.3000
5	LA D23 * −1	221.3060
6	LA D23 *	222.3120
7	Myristic acid (MA)	227.1990
8	MA +1	228.2040
9	MA +2	229.2090
10	MA D27 * −2	252.3560
11	MA D27 * −1	253.3620
12	MA D27 *	254.3690
13	Palmitic acid (PA)	255.2310
14	PA +1	256.2350
15	Stearic acid (SA)	283.2620
16	SA +1	284.2670
17	PA D31 * −1	285.4220
18	PA D31 *	286.4250
19	SA 13C18 * −1	300.3190
20	SA 13C18 *	301.3220

**Table 4:** Gradient for washing the column with 20 mM ammonium acetate as mobile phase A and methanol as mobile phase B. Water for mobile phase A can be used as well but was not in scope of method validation.

Block 1		Block 2 (Repeat Once More)		Block 3	
Time [min]	Mobile Phase B (Methanol) [%]	Time [min]	Mobile Phase B (Methanol) [%]	Time [min]	Mobile Phase B (Methanol) [%]
0.0	70.0	6.9	5.0	16.7	5.0
1.0	70.0	7.1	95.0	16.9	95.0
2.0	5.0	7.3	5.0	17.1	5.0
2.2	95.0	7.5	95.0	17.3	95.0
2.4	5.0	7.7	5.0	17.5	5.0
2.6	95.0	7.9	95.0	17.7	95.0
2.8	5.0	8.1	5.0	17.9	5.0
3.0	95.0	9.7	5.0	18.1	70.0
3.2	5.0	10.0	95.0	20.0	70.0
4.8	95.0	11.6	95.0		
5.1	5.0				
6.7	95.0				

**Table 5:** Gradient for column storage with 20 mM ammonium acetate as mobile phase A and methanol as mobile phase B. Water for mobile phase A can be used as well but was not in scope of method validation.

Block 1		Block 2	
Time [min]	Mobile Phase B (Methanol) [%]	Time [min]	Mobile Phase B (Methanol) [%]
0.0	70.0	7.6	95.0
2.0	5.0	7.8	5.0
7.0	5.0	13.5	5.0
7.2	95.0	14.5	100.0
7.4	5.0	20.0	100.0

**Table 6:** Summary table: Validation parameters with corresponding result performed by Hoelterhoff et al. [21]. Data adapted from Hoelterhoff et al. [21].

Validation Characteristic	Validation Result
Specificity	The method is able to separate, identify and determine the content of lauric, myristic, oleic, palmitic and stearic acid in protein samples. No interference (above Limit of Detection) with mobile phase A/B and w/o injection were detected.
Linearity	The coefficient of correlation for all tested fatty acids is greater than 0.99.
Accuracy by recovery	The recoveries of the 10 predefined levels from 50 to 40,000 ng/mL, after background subtraction, are all within 80–120%.
Precision	The relative standard deviation (per level) for all tested fatty acids is lower than 15%.
Repeatability	The relative standard deviation for all tested fatty acids is lower than 10%.
Intermediate Precision	The relative standard deviation for all tested fatty acids is lower than 10%.
Range	Lauric Acid: 0.05–40 µg/mL Myristic Acid: 0.05–40 µg/mL Oleic Acid: 0.1–40 µg/mL Palmitic Acid: 0.2–40 µg/mL Stearic Acid: 0.2–40 µg/mL
Limit of Quantitation (LOQ)	Lauric Acid: 49.7 ng/mL Myristic Acid: 49.7 ng/mL Oleic Acid: 103.5 ng/mL Palmitic Acid: 175.7 ng/mL Stearic Acid: 115.3 ng/mL
Limit of Detection (LOD)	Lauric Acid: 16.4 ng/mL Myristic Acid: 13.4 ng/mL Oleic Acid: 34.2 ng/mL Palmitic Acid: 55.3 ng/mL Stearic Acid: 38.0 ng/mL

## Data Availability

The datasets presented in this article are not readily available because of company guidelines.
